# Understanding the Impact of Age-Related Changes in Pediatric GI Solubility by Multivariate Data Analysis

**DOI:** 10.3390/pharmaceutics14020356

**Published:** 2022-02-04

**Authors:** Mariana Guimarães, Anil Maharaj, Andrea Edginton, Maria Vertzoni, Nikoletta Fotaki

**Affiliations:** 1 Department of Pharmacy and Pharmacology, University of Bath, Bath BA2 7AY, UK; mgsc1020@gmail.com; 2 Faculty of Pharmaceutical Sciences, The University of British Columbia, Vancouver, BC V6T 1Z3, Canada; anil.maharaj@ubc.ca; 3 School of Pharmacy, University of Waterloo, Waterloo, ON N2G 1C5, Canada; aedginto@uwaterloo.ca; 4 Department of Pharmacy, National and Kapodistrian University of Athens, 157 72 Athens, Greece; vertzoni@pharm.uoa.gr; 5 Centre for Therapeutic Innovation, University of Bath, Bath BA2 7AY, UK

**Keywords:** biopharmaceutics, pediatric, biorelevant, solubility, multivariate analysis

## Abstract

The aim of this study was to understand drug solubilization as a function of age and identify drugs at risk of altered drug solubility in newborns and young infants in comparison to adults. Multivariate statistical analysis was used to understand drug solubilization as a function of drug’s physicochemical properties and the composition of gastrointestinal fluids. The solubility of seven poorly soluble compounds was assessed in adult and age-specific fasted and fed state biorelevant media. Partial least squares regression (PLS-R) was used to assess the influence of (i) drug physicochemical properties and (ii) age-related changes in simulated GI fluids, as well as (iii) their interactions, on the pediatrics-to-adult solubility ratio (Sp/Sa (%)). For five out of seven of the compounds investigated, Sp/Sa (%) values fell outside of the 80–125% limits in at least one of the pediatric media. Lipophilicity was responsible for driving drug solubility differences between adults and children in all the biorelevant media investigated, while drug ionization was most relevant in the fed gastric media, and the fasted/fed intestinal media. The concentration of bile salts and lecithin in the fasted and fed intestinal media was critical in influencing drug solubility, while food composition (i.e., cow’s milk formula vs. soy formula) was a critical parameter in the fed gastric state. Changes in GI fluid composition between younger pediatric patients and adults can significantly alter drug luminal solubility. The use of pediatric biorelevant media can be helpful to identify the risk of altered drug solubilization in younger patients during drug development.

## 1. Introduction

Oral drug performance is dependent on oral drug bioavailability, the rate and extent of the drug reaching the systemic circulation unchanged. In addition to gut and hepatic metabolism, gastrointestinal (GI) absorption is a key factor influencing oral drug bioavailability. GI absorption is influenced by multiple factors, such as the extent and rate of drug dissolution, the formation of drug insoluble complexes, drug decomposition, and regional changes in drug permeability [1]. Only the drug that is in solution will be absorbed, and thus the magnitude of dissolved drug concentrations in the lumen influences the rate and extent of drug absorption [1]. Drug solubility in GI fluids dictates the upper concentration limit. Both intrinsic (e.g., anatomical, physiological) and extrinsic (e.g., nutritional) age-related changes affect the composition of GI fluids, which will ultimately impact drug solubility in the GI tract of pediatric patients [2]. There is a high rate of change in these intrinsic and extrinsic factors in the youngest subpopulations, especially during the first months of life [2].

The Biopharmaceutics Classification System (BCS) classifies drugs into four categories according to their solubility and permeability [3]. Drugs are assigned to one of the following categories: BCS class I (high solubility, high permeability), BCS class II (low solubility, high permeability), BCS class III (high solubility, low permeability) and BCS class IV (low solubility, low permeability). Over the years, the BCS has proved useful in several stages of adult drug development. For BCS class II and IV compounds, oral drug absorption will be limited by drug solubility. Age-related differences in GI fluids of pediatric patients in comparison to adults can affect drug solubility, and thus, alterations in drug performance can occur [4].

In the recent literature, the need for a Pediatric Biopharmaceutics Classification System (pBCS) has been highlighted [2]; its development will allow for a more simplistic way of understanding possible changes in oral drug performance in pediatrics. However, due to insufficient knowledge concerning the influence of growth and maturation on the GI tract, the establishment of a pBCS has not been possible so far.

According to the adult BCS, drugs are classified as highly soluble if the highest dose strength is soluble in 250 mL (or less) of aqueous liquid at a relevant physiological pH range of 1.2–6.8 [2,5,6]. Several issues arise concerning drug solubility classification within pediatrics, which include: (i) defining the highest single dose, (ii) Defining the initial gastric volume that is available upon drug arrival, and (iii) Establishing the luminal solubility of the drug; all of these factors vary amongst pediatric subpopulations, hindering the development of a pBCS [2,5,6].

Concerning drug permeability classification, drugs are classified as highly permeable when the extent of oral absorption (i.e., the fraction of dose absorbed) is greater than 85% of the administered dose [2,5,6]. If not available, correlations between in vivo and in vitro measurements of permeability are generally used, such as in vitro cell lines including Caco-2, or MDCK, as well as in silico models based on logP or logD data [4]. Due to the controversial nature of using adult permeability data for pediatric subjects, and the scarcity of information on permeability in newborns and infants, establishing meaningful permeability criteria for these subpopulations has not yet been possible [2,5,6].

The heterogeneity of the pediatric population poses another hurdle in the establishment of a pBCS, where balance is needed to develop a simple tool that discriminates the several pediatric subpopulations as much as possible. While such a tool remains to be developed, extrapolation of the adult BCS classification should be performed with care. The risk of using the BCS adult classification in pediatric drug development lies in possible shifts in the BCS classification of drugs due to alterations in the rate-limiting step of absorption resulting from growth and maturation [7]. For example, BCS class I and III drugs are eligible for the submission of BCS-based biowaivers to regulatory agencies, such as the European Medicines Agency (EMA) and the United States Food and Drug Administration (FDA). Therefore, extrapolation of drug product bioequivalence, in specific cases, can be based on in vitro dissolution experiments [4,6]. If age-related physiological and/or anatomical changes are responsible for shifts in the BCS classification as a result of differences in drug solubility and/or permeability classification, the BCS biowaiver status of a certain compound can be impacted for pediatrics.

In the field of biopharmaceutics, biorelevant media that simulate the composition of GI contents in adults are commonly used. Recently, age-specific biorelevant media have been proposed for newborns (0–28 days) and infants (1–12 months) [4]. The solubility of seven poorly water-soluble compounds was assessed in pediatric and adult biorelevant media to understand the impact of developmental differences in GI fluid composition on drug solubility.

This study aimed to understand drug solubilization as a function of age, and identify drugs at risk of altered drug solubility between newborns and young infants and adults. The solubility of seven drugs was assessed in adult and pediatric age-specific biorelevant media, in the fasted and fed state gastrointestinal (GI) simulated conditions. The characteristics of the investigated model drugs were restricted to include poorly water-soluble compounds, with a variety of physicochemical properties, and with documented usage in pediatrics and adults. Partial least squares regression (PLS-R) was used to evaluate the influence of drugs’ physicochemical properties and the composition of gastrointestinal fluids, as well as their interactions, on the pediatric-to-adult solubility ratio (Sp/Sa (%)).

## 2. Materials and Methods

### 2.1. Materials

Ultra-high-temperature-treated whole cow’s milk standardized to less than 4% of fat was acquired from Sainsbury’s, UK. The formula milk used in all experiments was First Infant Milk (cow’s milk-based formula) (Cow & Gate, Trowbridge, UK), and Wysoy (soy-based formula) (SMA-Nestlé, Gatwick, UK). Trifluoroacetic acid (TFA) (high-performance liquid chromatography (HPLC) grade), dimethyl sulfoxide (DMSO), sodium hydroxide, 37% hydrochloric acid, sodium chloride, sodium acetate trihydrate, ammonium acetate (HPLC grade), phosphate buffer salt, acetonitrile (I) (HPLC grade) and methanol (MeOH) (HPLC grade) were purchased from Fisher Scientific (UK). Montelukast sodium, mebendazole, mesalazine, sodium oleate, pepsin (from porcine), formic acid, maleic acid, dialysis tubing (12–14,000 Da molecular weight (MW) cut-off) and ammonium phosphate were obtained from Sigma-Aldrich Company Ltd., UK. Naproxen, amiodarone hydrochloride, nitrofurantoin and dipyridamole were obtained from VWR International, LLC. Water was ultra-pure (Milli-Q) laboratory grade. Sodium taurocholate (Prodotti Chimici Alimentari S.P.A., Basaluzzo, Italy), egg lecithin Lipoid EPCS (Lipoid GmbH, Ludwigshafen am Rhein, Germany) and glyceryl monooleate-Rylo Mg 19 (Danisco, Brabrand, Denmark) were obtained from the specified sources. Polytetrafluoroethylene (PTFE) filters (13 mm, 0.45 μm) were purchased from Fisher Scientific (Loughborough, UK).

### 2.2. Instrumentation

The equipment utilized in the investigation included a Buchi R114 Rotavapor (Switzerland), a Beckman Coulter J2-MC centrifuge (High Wycombe, UK), a pH meter (Mettler Toledo InLab Expert Pro-ISM, Schwerzenbach, Switzerland), a shaking water bath (model Grant SS40-2, Grant Instruments, UK), and a vortex mixer Rotamixer (East Grinstead, UK). HPLC analysis was performed using a Hewlett Packard Series 1100 system equipped with an autosampler, a temperature-regulated column compartment, a quaternary pump and a diode array detector (DAD) (Agilent Technologies, Santa Clara, CA, USA)

### 2.3. Drugs

The solubility of seven poorly water-soluble compounds (BCS class II and IV) was investigated, including three weak bases (dipyridamole, mebendazole and amiodarone), two amphoteric compounds (mesalazine and montelukast), and two weak acids (nitrofurantoin and naproxen). The physicochemical properties of the model drugs used are presented in Table 1.

The obtained results were combined with values from a previous study, where solubility in pediatric and adult media was obtained for seven additional poorly water-soluble compounds (neutral: griseofulvin, spironolactone, carbamazepine, and fenofibrate; weak acids: phenytoin and indomethacin; weak base: dapsone) [4].

### 2.4. Solubility Studies

Adult and pediatric biorelevant media were prepared according to Maharaj et al. [4]. Solubility experiments were performed using the shake flask method, in a shaking water bath (37 °C, 200 strokes/min) [4]. All solubility assessments were conducted in triplicate. An excess of the solid drug was used to saturate the volume of the medium used. In aqueous-based media (i.e., adult fasted state simulated gastric fluids (FaSSGF), adult fasted state simulated intestinal fluids (FaSSIF-V2) and adult fasted state simulated intestinal fluids (FeSSIF-V2)), experiments were conducted with 2 mL of medium in centrifuge tubes, and samples were collected at 24 h, media samples were filtered through 0.45 μm PTFE filters, diluted with fresh media and injected into the HPLC. For all the test drugs, filter adsorption studies were performed before the solubility measurements. Negligible drug adsorption to the filters used was observed (% drug adsorbed did not exceed 2%). In all media containing milk products (i.e., adult fed state simulated gastric fluids (FeSSGF)), the membrane dialysis technique was used to separate the undissolved drug. In this case, excess solid was added to the outside of the dialysis membrane. Experiments using milk-based media were conducted in 15 mL centrifuge tubes with 5 mL of the respective medium outside the membrane and 1 mL inside of the membrane. For sample collection, tubes were taken from the water bath, the dialysis membrane was removed, and its contents were collected at 48 h [4]. A longer dwell period was used for milk-based samples to ensure that the solubility results were not limited by drug diffusion through the dialysis membrane. After collection, the milk-based media solubility samples (media within the membrane) were immediately centrifuged in 1500 µL plastic centrifugation tubes (8000 rpm for 15 min at 4 °C). In this process, 1000 µL of extraction solvent (methanol for all drugs, except for mebendazole and nitrofurantoin, for which DMSO was used, and mesalazine, for which 0.01 % TFA was used) was added to 500 µL of the supernatant. The mixture was vortexed for 1 min and centrifuged at 8000 rpm for 15 min at 4 °C. The supernatant was filtered through a 0.45 µm PTFE filter and injected into the HPLC. 

### 2.5. Chromatographic Conditions for the Analysis of the Solubility Samples

Solubility samples were quantified using high-performance liquid chromatography with an ultraviolet detector (HPLC-UV). Analytical HPLC procedures were based on modifications of methods depicted in the literature and are denoted in Table 1. All calibration curves for aqueous-based media (FaSSGF, FaSSIF-V2, and FeSSIF-V2) were prepared with five standard concentrations created as mobile phase dilutions of a concentrated stock solution of the drug dissolved in a compatible organic solvent. Milk-based media standards were prepared in the same conditions as samples (i.e., created by dilution of the drug stock solution with fresh medium and treated as previously described). Stock solutions were prepared in methanol, except for mebendazole and nitrofurantoin, which were prepared in DMSO, and mesalazine, which was prepared in a solution of methanol:water with 0.01% TFA (5:95).

### 2.6. Treatment of In Vitro Solubility Data

Drug solubility differences between using pediatric media and the corresponding reference adult media were expressed as a ratio % (Sp/Sa (%)), where Sp is the solubility in each pediatric medium and Sa is the solubility in respective adult medium. Sp/Sa (%) values lower than 100% represent lower drug solubility in pediatric media when compared to adult reference media, and Sp/Sa (%) values higher than 100% represent the opposite. To denote relevant discrepancies in solubility between adults and pediatrics, biopharmaceutic risk assessment limits corresponding to reference values of 80 and 125% were set. Sp/Sa (%) ratios outside these limits were considered at a higher risk of altered drug performance between pediatric and adult patients.

Graphs comparing the pediatric-to-adult drug solubility ratio in biorelevant media (Sp/Sa (%)) ± SD were constructed in Microsoft Excel 2016, Office 365^®^ (Microsoft, London, UK). For the correlation of drugs’ physicochemical properties and solubility ratio (Sp/Sa (%)), 3D scatter plots and contour plots were generated. The 3D scatter plots conveyed Sp/Sa (%) as a function of logP and ionized (%), and were constructed using Minitab^®^ 19.2020.1 (^©^2020 Minitab, LLC, Coventry, UK). The contour plots portraying drug Sp/Sa (%) as a function of drugs logP and medium bile salts concentrations were generated using SigmaPlot 13.0 (SystatSoftware Inc., Slough, UK).

### 2.7. Statistical Analysis

All statistical evaluations were performed with XLSTAT^®^ add-in (Addinsoft, New York, USA) for Microsoft Excel 2016, Office 365^®^ (Microsoft, London, UK). 

One-way analysis of variance (ANOVA) with a post hoc Tukey’s multiple comparisons test was performed to assess statistical significance between drug solubility in each set of simulated fluids (i.e., neonate, infant and adult FaSSGF, etc.) using a significance level of *p* ≤ 0.05. 

**Figure 1:**
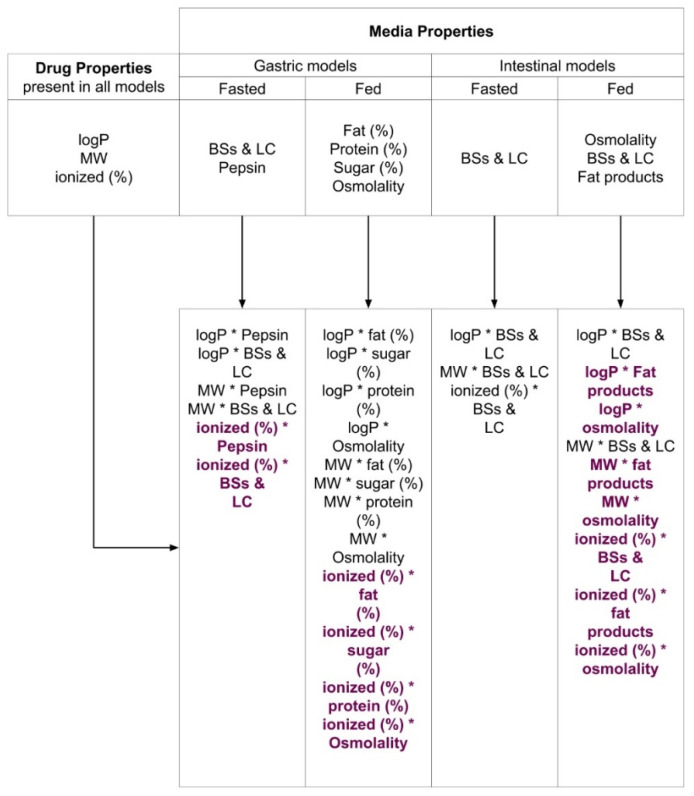
Variables present in each model investigated (fasted gastric; fed gastric; fasted intestinal and fed intestinal). Drug properties were maintained in all models, and media properties were selected according to changes between pediatric and adult media. Investigated interactions (between drug properties and media properties) are also described, where interactions colored in purple represent interactions with VIP values lower than 0.7.

Partial least squares regression (PLS-R) analysis was performed due to its ability to deal with collinearity between independent variables [24]. Sp/Sa (%) was set as the response variable for the PLS-R analysis. Explanatory variables were set as drugs’ physicochemical properties (i.e., logP, ionized (%) (positive value for compounds with a basic pKa, and a negative value for compounds with an acidic pKa, obtained from ACD/Labs^© 2022^–2018), and MW), changes in media components when comparing between pediatric and adult biorelevant media (i.e., pepsin, bile salts, lecithin, sodium oleate, glyceryl monooleate, fat (%), sugar (%), protein (%), etc.), and interactions between the medium and drug individual variables. A schematic of the independent variables and interaction terms used for each model is presented in Figure 1.

Composite variables (combination of two or more individual variables) were used when the ratio of the individual components for each of the composite variables was maintained throughout each pediatric medium. The influence of “bile salts and lecithin (BSs and LC)” was evaluated as a composite variable comprised of sodium taurocholate (NaTC) and lecithin concentrations, and the variable “fat products” was set to represent concentrations of sodium oleate and glyceryl monooleate. The PLS-R analysis was conducted for the seven compounds investigated in this study and for an additional seven compounds investigated in a previous study [4]. The PLS-R regression generates components based on the defined independent variables to explain the response [25]. The number of components will be lower than the initial independent variables selected for the PLS-R models. The PLS-R components are built iteratively so that they explain as well as possible the variability of Y (also known as the response and dependent variable). The model quality was evaluated based on the square of the coefficient of determination (R^2^) and goodness of prediction (Q^2^), where R^2^ and Q^2^ close to 1 represent good model fit and high predictive power, respectively [26]. The number of principal components for each model was selected based on the model’s optimum Q^2^ value and predicted residual error sum of squares (PRESS) [24,25,27]. A Q^2^ value higher than 0.5 is considered acceptable for good model predictability [26]. The PLS-R was built and evaluated based on full cross-validation (leave-one-out procedure). Independent variables were considered to have an impact on the response if variable importance in projection (VIP) values were higher than 1.0, whereas 0.7–1.0 values were regarded as important (i.e., moderate impact), and values lower than 0.7 were generally regarded as insignificant [24,25,27]. Standardized coefficients were generated for each independent variable and selected interactions. These coefficients were related to the independent variables and the response variance and were normalised for the variation of Y. The normalisation of the coefficients was accomplished by dividing the non-standardized coefficient value obtained for each independent variable by its respective standard deviation (SD) [25]. In the PLS-R analysis, the generation of standardized coefficients allowed the comparison between variables within a model but not between different models [25]. Outliers in the analyses were identified by means of the comparison of the distance of each observation to the model in the Y-plane (DModY) with their calculated critical value (DCrit(Y)); an outlier was present if the standardized DCritY for an observation was greater than DCritY [28,29,30]. Outliers were only excluded if (i) the PLS model improved significantly after exclusion and (ii) there was a clear scientific rational on why the data should be excluded (i.e., why the current models would not be able to explain the results).

## 3. Results

### 3.1. Drug Solubility Assessments

Drug solubility values in adult reference media (i.e., FaSSGF, FeSSGF, FaSSIF-V2 and FeSSIF-V2) are presented in Table 2.

**Figure 2:**
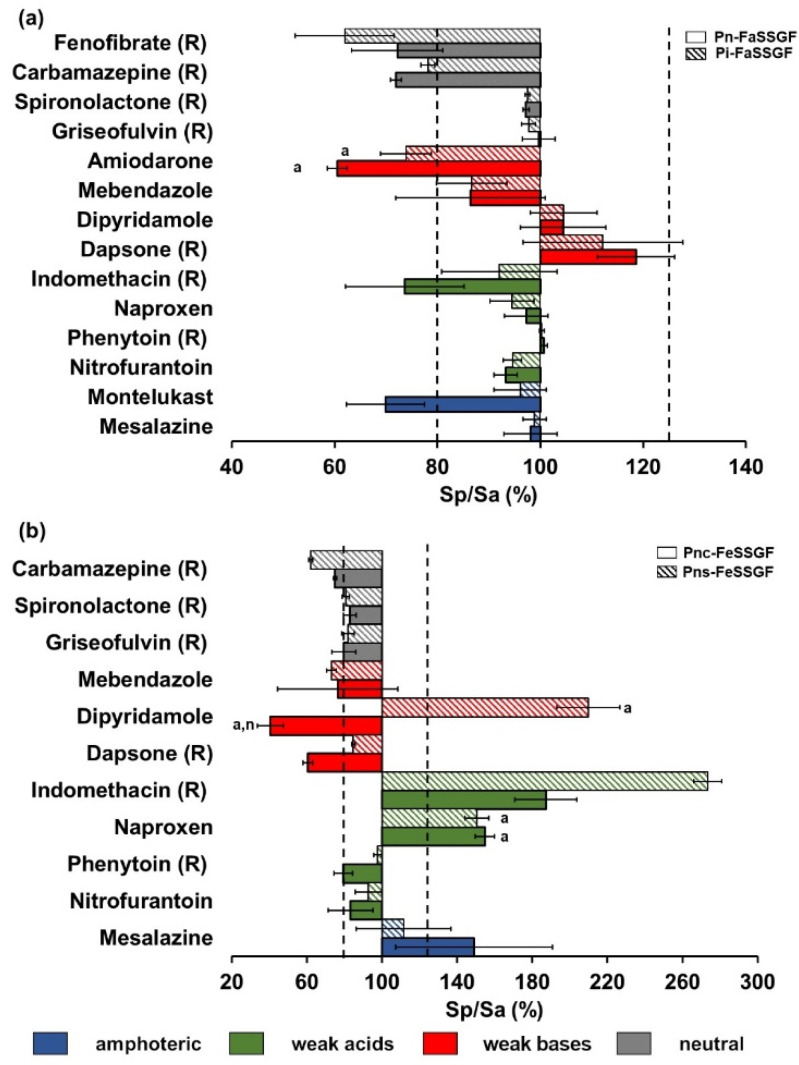
Pediatric-to-adult drug solubility ratio in biorelevant media (Sp/Sa (%)) ± SD in (**a**) fasted and (**b**) fed gastric simulated fluids. The solubility of compounds noted with (R) was investigated in a previous study and is displayed for reference [4]. Dotted lines represent the 80–125% boundary. Statistically significant solubility differences (*p* ≤ 0.05) compared to a—the adult medium, and n—the newborn medium are denoted by the indicated letters. Media are denoted as follows: Pi-FaSSGF (infant FaSSGF), Pn-FaSSGF (newborn FaSSGF), Pnc-FeSSGF (newborn FeSSGF containing cow’s milk-based formula), Pns-FeSSGF (newborn FeSSGF containing soy-based formula).

**Figure 3:**
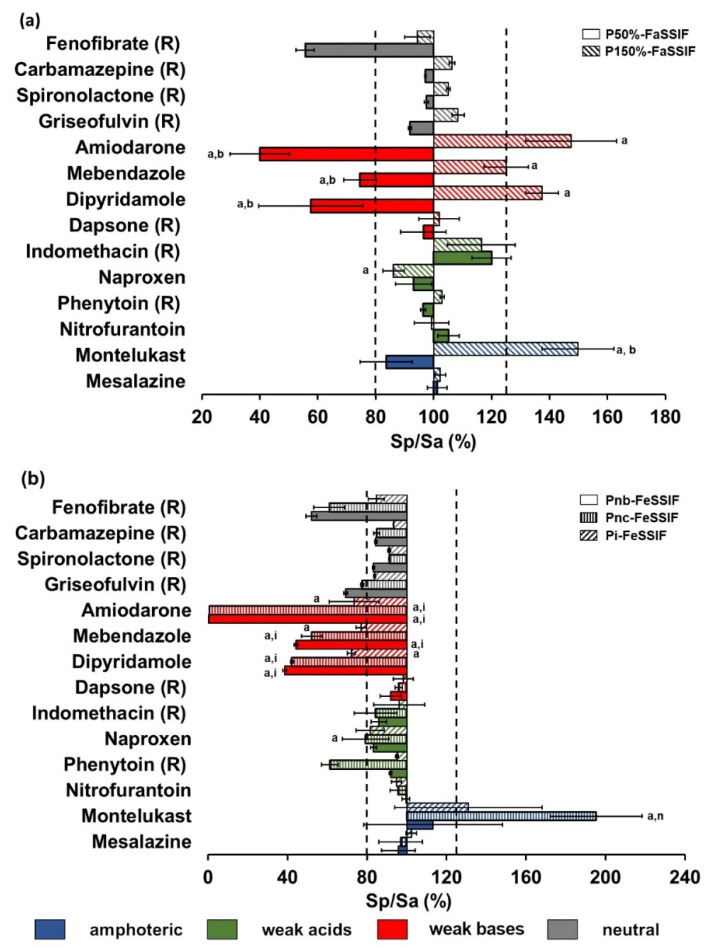
Pediatric-to-adult solubility ratio in biorelevant media (Sp/Sa (%)) ± SD in (**a**) fasted and (**b**) fed intestinal simulated fluids. The solubility of the compounds noted with (R) was investigated in a previous study and is displayed for reference [4]. Dotted lines represent the 80–125% boundary. Statistically significant solubility differences (*p* ≤ 0.05) compared to a—the adult medium, i—the infant medium, n—the newborn medium, and b—P150%-FaSSIF medium are denoted by the corresponding letters. Media are denoted as follows: P150%-FaSSIF (pediatric FaSSIF comprised with 4.5 mM NaTC), P50%-FaSSIF (pediatric FaSSIF comprised with 1.5 mM NaTC), Pi-FeSSIF (Infant FeSSIF), Pnb-FeSSIF (Newborn breast-fed FeSSIF), and Pnc-FeSSIF (Newborn formula-fed FeSSIF).

Solubility results measured in each pediatric medium vs. solubility in the respective adult medium are expressed as the ratio Sp/Sa (%) (Figure 2 and Figure 3). In the following sections, differences in solubility in pediatric media in comparison to adult media will be explored. 

#### 3.1.1. Fasted Gastric Simulated Fluids

Two pediatric media simulating the fasted gastric fluids of newborns (Pn-FaSSGF) and infants (Pi-FaSSGF) were tested. Lower concentrations of pepsin, bile salts and lecithin are present in the pediatric media in comparison to the adult respective medium. The pH of adult and pediatric media fasted gastric simulated fluid is 1.6. The compositions of the adult and pediatric biorelevant media were as described by Maharaj et al. [4]. Six of the seven compounds (all except dipyridamole, a weak base with logP of 2.7) exhibited lower solubility in at least one of the pediatric gastric media (Pn-FaSSGF and Pi-FaSSGF). For the amphoteric compound montelukast (pKa of 2.7 (basic) and 5.8 (acidic) and logP 8.8), mean Sp/Sa (%) was outside the 80–125% threshold (Figure 2a) in the newborn’s gastric simulated fluids.

However, no statistically significant differences were observed between the solubility in the different fasted gastric media investigated. Among the weak acids investigated in this study, solubility was similar between all the tested adult and pediatric fasted gastric fluids. In a previous study, Sp/Sa (%) fell outside the limits of the 80–125% threshold for indomethacin (weak acid, logP 4.3) in the newborn gastric media (Pn-FaSSGF). Among the weak bases investigated in this study, amiodarone (logP 7.6) showed differences in mean Sp/Sa (%) in both pediatric fasted gastric fluids that fell outside the 80–125% threshold (Figure 2a). These solubility values were statistically different in comparison to the solubility values in the reference adult media (*p* ≤ 0.05). Dipyridamole was the only weak base that showed higher solubility in both pediatric gastric fluids; however, these differences were not statistically significant. 

All neutral compounds were investigated in a previous study which showed that carbamazepine (logP 2.5) and fenofibrate’s (5.3) solubility values in pediatric gastric fluids in comparison to adult gastric fluids were both statistical different (*p* ≤ 0.05) and outside the purported bioequivalence criterion [4]. These results suggest that high lipophilic compounds (logP > 4) are at a higher risk of alterations in drug solubility.

#### 3.1.2. Fed Gastric Simulated Fluids

Pediatric media simulating fed gastric conditions (Pnc-FeSSGF and Pns-FeSSGF) display different composition from the corresponding adult medium in terms of buffer capacity, osmolality, pH (pH of adult medium: 5; pH of pediatric media: 5.7), and type of mil’ (cow’s milk formula and soy formula). The composition of the adult and pediatric biorelevant media were as described by Maharaj et al. [4]. Equilibrium dialysis was used to separate undissolved drugs in fed state gastric media (all milk-based media) to ensure consistency with the previous solubility study in pediatric media [4]. However, the technique was found to be unsuitable for montelukast and amiodarone. This assertion was based on the use of a confirmation technique, centrifugation, followed by protein precipitation of the supernatant and sample treatment for the extraction of the dissolved drug, performed for adult fed gastric medium (FeSSGF). The solubility of montelukast and amiodarone by equilibrium dialysis was ~10-fold below the observed solubility using centrifugation, and samples presented high variability; therefore, results are not presented in this study. Four drugs out of the seven drugs tested in this study exhibited Sp/Sa (%) outside of the 80–125 % limits for at least one of the newborn fed gastric media (Figure 2b). For the amphoteric compound mesalazine (pKa of 2.3 (acidic) and 5.7 (basic) and logP 0.98), solubility in both pediatric media were not significantly different (*p* ≥ 0.05) from drug solubility values in adult biorelevant medium (FeSSGF), nor when comparing solubility values between the two pediatric medium (Pnc-FeSSGF and Pns-FeSSGF). The adult medium representative of fed gastric fluids (FeSSGF) presents a pH of 5, whereas both pediatric media simulating newborns fed with cow’s milk and soy formula (Pnc-FeSSGF and Pns-FeSSGF, respectively) have a pH value of 5.7. Thus, the differences observed for ionizable compounds are, in part, related to higher or lower ionization in the pediatric media, based on the drug ionization properties. Among the weak acid compounds tested, naproxen (logP of 3.2) showed significantly higher solubility in both pediatric media when compared to solubility in adult media. Naproxen Sp/Sa (%) was also outside the 80–125% limits set in this study for both pediatric media (containing cow (Pnc-FeSSGF) and soy formula milk (Pns-FeSSGF)). This can be attributed to the ionization state of the compound, since naproxen has an acidic pKa of 4.18, and therefore shows a higher ionized (%) in both media reflective of newborn conditions in the fed state. 

Among the weak bases tested, mebendazole (logP of 2.8) showed lower solubility in both pediatric media (Pnc-FeSSGF and Pns-FeSSGF); however, these differences were not significant when compared to solubility in adult media and where not outside the 80–125% limits for the Sp/Sa (%). For the weak base dipyridamole, significantly higher solubility values in Pns-FeSSGF (medium containing soy formula) were observed in comparison to solubility in adult media; conversely, significantly lower solubility was observed in Pnc-FeSSGF (medium containing cow’s milk formula). These results suggest that different types of feeding may lead to changes in drug solubility. The solubility of dipyridamole has been previously measured in human gastric fluids aspirates after administration of 500 mL of Ensure plus to healthy adults [31], and it was shown that differences in GI fluid composition as a function of time after food ingestion influenced the solubility of dipyridamole [31].

In a previous study, Sp/Sa (%) fell outside the limits of the 80–125% for five drugs (two neutral compounds: carbamazepine, griseofulvin; one weak base: dapsone; and two weak acids: phenytoin, and indomethacin) for at least one of the newborn media. 

#### 3.1.3. Fasted Intestinal Simulated Fluids

Literature review of pediatric bile salt concentration in the fasted intestine revealed high variability. To explore the impact of variability changes in bile salts concentration in pediatric luminal fluids, two pediatric media were developed by Maharaj et al. [4]. Consequently, media with 50% and 150% of bile salt (NaTC) and lecithin values of the respective adult biorelevant medium (FaSSIF-V2) were used in this study [4]. Note that the pH of both adult and pediatric fasted intestinal simulated fluids is 6.5, as previously described [4]. 

When compared to adult fasted state intestinal simulating fluids (FaSSIF-V2), drug solubility in pediatric media fell outside of the threshold of 80–125% for four drugs (Figure 3a). Five of the seven compounds studied showed lower solubility when bile salts and lecithin concentrations were decreased (medium with 50% [NaTC] = 1.5 mM) and higher solubility values were observed when bile salts were increased (medium with 150% [NaTC] = 4.5 mM). 

For the amphoteric compound montelukast (pKa of 2.7 (basic) and 5.8 (acidic) and logP 8.8), a statistically significant solubility difference was found in the pediatric fluid, simulating higher concentrations of bile salts (P150%-FaSSIF) when compared to adult fasted intestinal medium (FaSSIF-V2). 

Among the weak acids, naproxen did not follow the general trend observed and showed significantly lower solubility in P150%-FaSSIF when compared to adult FaSSIF-V2; however, the Sp/Sa (%) was within the 80–125% boundaries.

For the weak bases amiodarone, dipyridamole, and mebendazole, solubility was significantly different (*p* ≤ 0.05) in both pediatric fasted state intestinal media (P50%-FaSSIF and P150%-FaSSIF) when compared to the reference adult medium (FaSSIF-V2). 

In the previous solubility study, Sp/Sa (%) fell outside the 80–125% threshold for six of the seven compounds (all except dapsone, a weak base with logP of 0.97) in both pediatric media; moreover, statistically significant drug solubility differences (*p* ≤ 0.05) were also observed in at least one of the pediatric FaSSIF media in comparison with drug solubility in adult FaSSIF-V2 [4].

#### 3.1.4. Fed Intestinal Simulated Fluids

Maharaj et al. developed pediatric media to simulate the intestinal fluids of breastfed newborns (Pnb-FeSSIF), newborns fed with cow’s milk formula (Pnc-FeSSIF) and infants fed with cow’s milk formula (Pi-FeSSIF) [4]. Differences in pediatric fed state intestinal simulating fluids were designed to simulate the decreased concentrations of bile salts, lecithin, sodium oleate, glyceryl monooleate, and osmolality. The pH of adult and pediatric fed intestinal simulated fluids is 5.8, as previously described [4]. 

Four out of seven compounds showed Sp/Sa (%) values that fell outside the 80–125% threshold (Figure 3b). Six out of the seven compounds displayed lower solubility in at least one of the pediatric fed state intestinal media (Pnb-FeSSIF, Pnc-FeSSIF, and Pi-FeSSIF), likely due to the lower concentrations of bile salts and lecithin.

Among the amphoteric compounds tested in this study, montelukast (pKa of 2.7 (basic) and 5.8 (acidic) and logP 8.8) showed significantly higher solubility in Pnc-FeSSIF when compared to Pnb-FeSSIF and adult FeSSIF-V2. Moreover, the pediatric-to-adult solubility ratios calculated for montelukast were outside of 80–125% limits for two of the pediatric media (Pnc-FeSSIF and Pi-FeSSIF).

For the weak acids tested in this study, Sp/Sa (%) values were outside the 80–125% threshold in the pediatric medium Pnc-FeSSIF, and solubilities were significantly lower when compared to the adult FeSSIF-V2.

For all weak bases tested in this study (amiodarone, mebendazole, and dipyridamole), significant solubility differences were observed in all pediatric media (Pnb-FeSSIF, Pnc-FeSSIF, and Pi-FeSSIF) when compared to the reference adult medium (FeSSIF-V2). These compounds also showed differences between their solubility in newborn and infant fed state intestinal media (Pnb-FeSSIF and Pnc-FeSSIF vs. Pi-FeSSIF). Amiodarone (logP of 7.6) solubility was much lower in pediatric media when compared to the adult medium. The solubility of amiodarone has been previously shown to be very sensitive to changes in lecithin concentrations present in biorelevant media [21].

In the previous solubility study, Sp/Sa (%) for three compounds (fenofibrate, griseofulvin, and phenytoin) were below 80% of adult values in at least one of the pediatric newborn FeSSIF (Pnb-FeSSIF and Pnc-FeSSIF) [4]. With regard to the infant medium (Pi-FeSSIF), Sp/Sa (%) fell within the 80–125% threshold of adult values for all compounds investigated.

### 3.2. Correlation of Drugs’ Physicochemical Properties and Solubility Ratio

**Figure 4:**
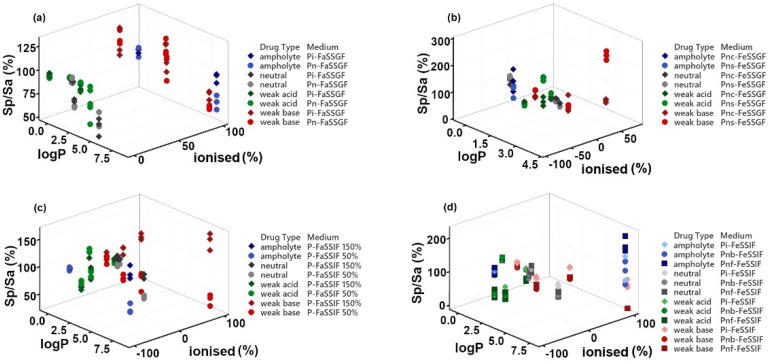
Three-dimensional scatter plot of mean pediatric-to-adult solubility ratio (Sp/Sa (%)) as a function of logP and ionized (%) for the: (**a**) fasted gastric simulated fluids; (**b**) fed gastric simulated fluids; (**c**) fasted intestinal simulated fluids; (**d**) fed intestinal simulated fluids.

The 3D correlations of Sp/Sa (%) vs. drug logP and ionized (%) for the fasted and fed state of the GI tract are presented in Figure 4. A trend of decreasing Sp/Sa (%) with increasing lipophilicity can be observed, regardless of the ionization state in the fasted gastric pediatric fluids (Figure 4a). The lowest pediatric-to-adult solubility ratios (Sp/Sa (%)) were seen for indomethacin (weak acid) and amiodarone (weak base), which are the most lipophilic compounds from their respective groups, with a logP of 4.3 and 7.6, respectively.

In pediatric fed gastric simulated fluids, a trend was observed for the compounds with higher negative ionized (%) to display a higher Sp/Sa (%) (Figure 4b). 

In the fasted state intestinal fluids, a general trend was observed for the effect of the increase in bile salts and lecithin concentrations on Sp/Sa (%), specifically for weak bases with a higher ionized (%) (i.e., higher than 40%) and/or with high lipophilicity (i.e., higher than logP = 3) (Figure 4c); consequently, higher differences between adult and pediatric solubility were observed for weak bases in pediatric FaSSIF. In fed state simulated intestinal fluids (Figure 4d), differences between pediatric and adult solubility were observed for ionized and non-ionized compounds. Larger differences between pediatric and adult solubility (reflected as Sp/Sa (%) distant from 100%) were visualized for weak bases when compared to weak acids and neutral drugs; moreover, lower solubility in pediatric fed intestinal fluids was observed as drug logP increased. 

**Figure 5:**
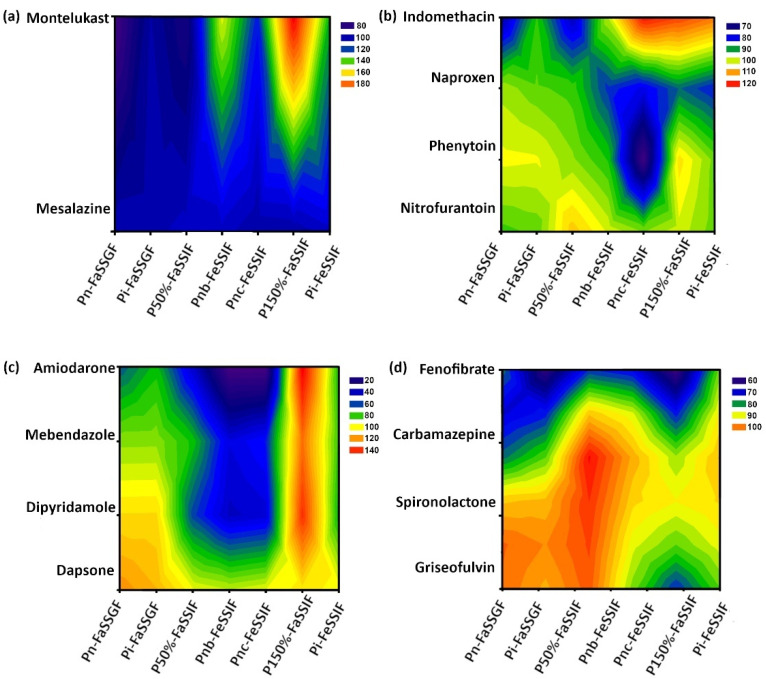
Contour plots of average pediatric-to-adult solubility ratio (Sp/Sa (%)) according to rank-ordered logP (drugs were ordered as a function of logP from −0.47 to 9) and rank-ordered by the concentration of bile salts in each pediatric medium (ordered from the medium with lower amounts of to higher amounts of NaTC): (**a**) ampholyte compounds; (**b**) weak acids; (**c**) weak bases; and (**d**) neutral.

The correlation between pediatric and adult solubility ratios (Sp/Sa (%)) and the medium bile salts (NaTC) concentrations and logP is presented in Figure 5. For montelukast (logP of 8.8), a general trend was identified for an increase in Sp/Sa (%) with the increasing bile salts concentrations (Figure 5a). 

All weak bases (Figure 5c) were affected by the concentration of bile salts, with a trend for higher Sp/Sa (%) in media with a higher content of bile salts observed. Additionally, the lipophilicity of weak bases appears to be correlated to the level of impact of changes in bile salt concentrations on drug solubility. In comparison, only extremely lipophilic weak acids appeared to be affected by changes in medium bile salt concentrations, and no major changes in Sp/Sa (%) were observed for compounds with logP values below 5 (Figure 5b). The exception to this was phenytoin (weak acid with moderate logP of 2.47), for which a significant lower Sp/Sa (%) was observed in Pnc-FeSSIF, which could be related to the fact that in this medium, there were not only changes in NaTC, but also differences in the concentration of fat digestion products. 

For neutral compounds (Figure 5d), except for fenofibrate and carbamazepine, no differences were observed between pediatric and adult drug solubility values. For fenofibrate, the Sp/Sa (%) was always lower than 80 % in all bile salt concentration levels.

### 3.3. Multivariate Statistical Analysis

#### 3.3.1. Fasted Gastric Simulated Fluids

**Figure 6:**
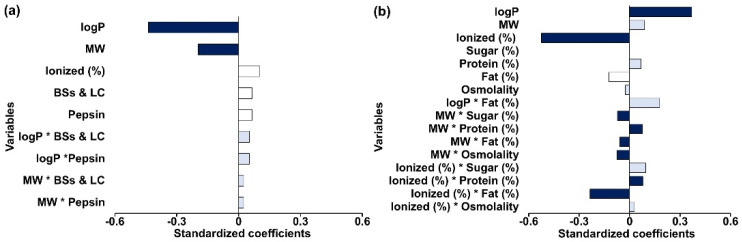
Standardized coefficients in (**a**) fasted and (**b**) fed pediatric gastric fluids corresponding to the variables investigated in each model. Blue dark color denotes coefficients of VIP ≥ 1.0 (significant impact), and light blue color represents variables with VIP ≥ 0.7 (moderate impact), which are considered to have an effect on the solubility ratios between pediatric and adult media (Sp/Sa (%)).

The PLS-R model for the pediatric fasted gastric conditions was constructed using Sp/Sa (%) in newborn and infant media (Pn-FaSSGF and Pi-FaSSGF) as the dependent variable and the independent variables, as described in Figure 1. The model was best defined by two principal components and accounted for a low percentage of variability of the dependent variable (R^2^ = 0.37) and a limited predictive power (Q^2^ = 0.25). The model depicted that lipophilicity (logP) and MW were the most important factors (VIP higher than 1) affecting Sp/Sa (%). Negative standardized coefficient values associated with drug logP and MW (Figure 6a) indicate that these variables showed a negative effect on the Sp/Sa (%). Interactions between logP or MW and media properties (BSs and LC and pepsin concentrations) showed VIP values higher than 0.7, indicating a moderate potential for describing differences between pediatric and adult solubility values.

#### 3.3.2. Fed Gastric Simulated Fluids

The PLS-R model of drug solubility differences in the pediatric fed gastric state was best described by two principal components and showed an R^2^ of 0.75 and acceptable predictive power (Q^2^ = 0.56). The model showed that drug ionized (%) and lipophilicity (logP) are the two single factors with the highest effect on Sp/Sa (%) (VIP ≥ 1). Based on standardized coefficient values (Figure 6b), logP, MW, and ionized (%) were negatively correlated with pediatric-to-adult solubility ratios Sp/Sa (%). Ionized (%) was the variable with the highest absolute standardized coefficient value, indicating that it has the highest effect on the Sp/Sa (%). Additionally, interactions between ionized (%) or MW and meal properties (such as fat and protein content) significantly affected the pediatric-to-adult solubility ratio (%), indicating the complex interplay of drug and media properties on influencing drug solubility in pediatric GI fluids. 

#### 3.3.3. Fasted Intestinal Simulated Fluids

**Figure 7:**
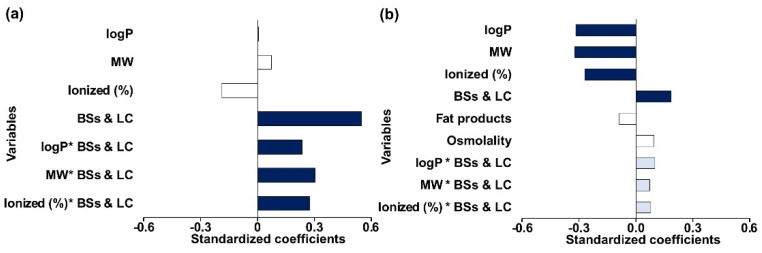
Standardized coefficients in (**a**) fasted and (**b**) fed pediatric intestinal fluids corresponding to the variables investigated in each model. Blue dark color denotes coefficients of VIP ≥ 1.0 (significant impact), and light blue color represents variables with VIP ≥ 0.7 (moderate impact), which are considered to affect the solubility ratios between pediatric and adult media (Sp/Sa (%)).

The PLS-R model for the Sp/Sa (%) in the fasted intestinal state achieved optimum Q^2^ with only one principal component and showed a good fit to the experimental data (R^2^ = 0.73) and good predictive power (Q^2^ = 0.61). The model revealed that BSs and LC was the only significant single factor influencing the solubility between pediatrics and adults (Sp/Sa (%)) (Figure 7a). Based on the standardized coefficient values, BSs and LC is positively correlated to the solubility differences between adults and pediatrics. All interaction terms with BSs and LC also showed a positive effect on the solubility ratio (Sp/Sa (%)), which suggests that drugs with higher lipophilicity, high molecular weight, and with ionizable properties are at a higher risk of showing solubility differences between adults and pediatrics if differences in bile salts concentrations are observed.

#### 3.3.4. Fed Intestinal Simulated Fluids

The PLS-R model developed for the Sp/Sa (%) in the fed state intestinal media showed good fit to the experimental data (R^2^ = 0.74) and good predictive power (Q^2^ = 0.79), using one principal component. The model showed that drug logP, MW and ionized (%), and BSs and LC were the most significant factors influencing Sp/Sa (%) (Figure 7b). Based on the standardized coefficient values, BSs and LC displayed a positive influence on the Sp/Sa (%), while drug lipophilicity (logP), ionized (%) and MW showed a negative effect on Sp/Sa (%). Interactions of the drug properties with BSs and LC were moderately important (VIP > 0.7) and displayed a positive effect on Sp/Sa (%). Note that montelukast observation in the fed intestinal media was identified by preliminary analysis as an outlier (data not shown) and excluded from the PLS-R analysis for the fed intestinal media. This is related to the fact that Sp/Sa (%) in fed state intestinal fluids showed values lower than 100% for all drugs, except for montelukast. The model quality (R^2^ and Q^2^) improved notably after the exclusion of montelukast. We hypothesize that this is related to the fact that for certain drugs, solubility changes in pediatrics when compared to adults may not be fully predicted by the investigated drug properties and/or media properties and might be related to other aspects that were not considered by the model. 

## 4. Discussion

Differences in drug solubilization between pediatric and adult biorelevant media were observed in the fasted and fed state gastrointestinal conditions. Of the compounds investigated in this study (n = 7), nitrofurantoin (weak acid, logP −0.47) was the only one for which solubility differences between pediatric and adult biorelevant media were within the 80–125% risk assessment limits for all pediatric media tested. Additionally, no significant solubility differences were found between adults and pediatrics. These results are probably related to nitrofurantoin’s low lipophilicity (logP = −0.47) and its acidic pKa of 7.2, meaning that it is primarily non-ionized in all the biorelevant media tested. 

For the fasted gastric state, a trend was identified for solubility ratios (Sp/Sa (%)) lower than 100%, which is in accordance with what was previously found [4]. The solubility differences in the fasted state gastric media seemed to be related to compound lipophilicity, as three of the compounds that show the highest differences have a logP > 5 (fenofibrate, montelukast and amiodarone). These observations are further confirmed by the PLS-R analysis which showed that logP and MW were the most important factors responsible for solubility differences between pediatric and adult media (Figure 6). The fact that these variables display a negative effect on Sp/Sa (%) indicates that there is a potential risk for reduced drug solubility of high lipophilic/high MW compounds in the younger pediatric patients. Note that the fasted gastric model accounted for a low degree of variability of the response (R^2^ = 0.37) and showed limited predictive power (Q^2^ = 0.25), therefore, caution is granted for interpretation of the results. 

For the fed gastric state, drug lipophilicity and ionization were shown to have a significant effect on the solubility differences between pediatric and adult fluids (Figure 6b) (7). Drug lipophilicity has previously been shown to play a role in the prominence of the impact of pH changes on drug solubility [32,33,34]. Taking into account that solubility is highly driven by pH changes and that the pediatric fed gastric media is the only set of media with differences in pH when compared to the respective adult medium, changes in drug solubility in these media are expected to be more significant for ionized compounds. In the PLS-R analysis, a significant effect of ionized (%) on the Sp/Sa (%) (positive or negative) is related to the ionization properties of the compound (weak acidic/weak basic pKa). For example, in the fed gastric state, higher differences between solubility in adults and pediatrics were observed for weak acids (specifically naproxen and indomethacin). For the majority of the weak bases, the opposite trend was observed; however, for dipyridamole (logP 2.74), a significantly higher solubility in Pns-FeSSGF (medium containing soy formula) and a significantly lower solubility in Pnc-FeSSGF (medium containing cow’s milk formula) were observed when compared to adult solubility in FeSSGF. Dipyridamole solubility in fed gastric pediatric media suggests that drugs might exhibit distinct affinities for infant cow’s milk or soy formula due to small differences in their composition, such as protein type, protein content, sugar content, and fat content. This observation is supported by the PLS-R results, which show for example that the interaction of drug ionized (%) and the protein and fat medium composition is a significant factor affecting Sp/Sa (%). The results from the statistical analysis show that differences in the composition between different types of milk products (e.g., formula, cow’s milk) can affect the solubility of drugs in GI fluids. Infant formula is developed to closely represent breast milk concerning the proportions of energy provided by macronutrients (proteins, fats and carbohydrates); however, differences with regard to fat (e.g., individual components of the lipid system such as triglycerides and phospholipids), protein type (whey and casein protein from milk vs. protein concentrate from soy), and whey to casein ratios of protein can be observed [4]. In this study, Wysoy^®^ by SMA^®^ and First Infant Milk by Cow & Gate^®^ were used as soy-based formula and cow’s milk-based formula, respectively, although protein content is similar for both formulas (1.8 g and 1.3 g per 100 mL of prepared formula, respectively), the type of protein is different. While the soy-based formula is composed of soy protein isolate, in cow’s milk-based formula, whey (0.8 g) and casein proteins (0.5 g) are present. Furthermore, while total carbohydrate content is similar (6.9 g in soy-based formula and 7.2 g in the cow-based formula), only 2.5 g of sugar is present in Wysoy^®^ compared to 7.2 g of sugar in First Infant Milk^®^, of which 7.0 g is lactose. It should be noted that lactose is not present in Wysoy^®^. 

In this study, the use of equilibrium dialysis for amiodarone (logP = 7.90) and montelukast (logP = 8.79) was challenging, and the results are not presented. Amiodarone and montelukast are extremely lipophilic (logP 7.6 and 8.8, respectively), with high MW (645 and 586, respectively), and also show high binding to plasma proteins (~97% and ~99%, respectively, according to ACD/I-Lab©, 2010–2018 ACD/Labs). Drug separation by equilibrium dialysis was also found to be ineffective in the previous study by Maharaj et al. [4]. For fenofibrate, a compound with very similar characteristics, and for which hemodialysis is considered inefficacious [4], if the drug is highly bound to the milk proteins and fat globules, this could limit the diffusion of the solubilized drug through the membrane. Limitations of equilibrium dialysis have previously been reported during drug release studies [35,36]. Drug adsorption to the dialysis membrane has also been reported as a limiting factor of this technique [37]. Most of these issues could be potentially resolved if enough time is given for diffusion to occur; however, because this study aimed to compare results between drugs, it was key that the dwell period was the same for all drugs.

In the fasted intestinal state, higher differences were found between pediatric media and the corresponding adult medium for the most lipophilic of the investigated compounds (montelukast, amiodarone and fenofibrate, for which logP is between 5 and 8). Sp/Sa (%) for 5 out of the 14 investigated compounds were outside the biopharmaceutical risk assessment limits. It appears that weak bases were the compounds most prone to be affected by changes in the concentrations of BSs and LC. The statistical analysis confirmed this interpretation and showed that BSs and LC is the only individual variable influencing solubility differences (positive impact). Additionally, interactions between BSs and LC and drug properties contribute towards defining the extent and magnitude of observed drug solubility changes. Drug lipophilicity, MW and ionization showed a positive impact on Sp/Sa (%), and therefore with higher BSs and LC concentrations and ionizable drugs with high logP or high MW, higher solubility ratios are expected (i.e., increased drug solubility in pediatric media in comparison to drug solubility in adult media). 

Sp/Sa (%) in fed state intestinal fluids showed values lower than 100% for all drugs, except montelukast. For this drug, a trend for higher Sp/Sa (%) was observed in all pediatric FeSSIF, although only the solubility in Pnc-FeSSIF was significantly different from adults (*p* ≤ 0.05). This is probably related to the fact that pediatric media contain a smaller amount of sodium chloride, and therefore the solubility results are probably related to a common-ion effect, since montelukast sodium was used in the experiments [38]. Pnc-FeSSIF medium simulates newborns intestinal conditions following feeding with cow’s milk formula, which contains the highest content of fat digestion products (sodium oleate and glyceryl monooleate); this can most likely explain the higher solubility of montelukast (logP = 8.8) in comparison to the remaining fed state intestinal media. 

The results of this study suggest that weak bases are at a higher risk of altered drug solubility in fasted and fed intestinal fluids, as shown by the impact of ionization as a single variable and/or the interaction of ionized drugs with BSs and LC. Additionally, logP and MW were also critical in modulating pediatric solubility either as individual variables or as interactions with BSs and LC. The results observed in the fasted and fed intestinal PLS-R analyses (positive effect of BSs and LC on drug solubility changes between adults and pediatrics) are in agreement with previous literature reports where the concentration of physiologically relevant surfactants (i.e., sodium oleate, bile salt and lecithin) were revealed to be more important than other components of biorelevant media, such as salt, buffer, and pancreatin, in modulating solubility in both fasted and fed intestinal fluids [32,33,34]. In accordance with this, Mithani et al. previously showed that the solubilization effect of bile salts (NaTC) increases with increasing drug lipophilicity, and that logP and MW can be used as a surrogate for predicting solubility as a function of bile salts concentrations [39]. Drug MW was selected as an independent variable in the models (Figure 7) for the investigation of drugs’ physicochemical properties, due to its relationship with the volume/space that the drug occupies, which can influence the way it will solubilize into micelles [40]. Even though drug logP and MW often display collinearity, they represent different properties of a molecule. For example, during dissolution studies, drug diffusion through a medium containing micelles will be influenced by the molecular weight of the drug, where larger drug-micelle complexes will migrate slower [41]. 

Due to the nature of the compounds selected for this study (poorly water-soluble, BCS class II and IV), age-related changes in drug solubility are more likely to play a role in influencing drug absorption. For BCS class II compounds, absorption is highly influenced by the solubility and the rate of drug dissolution in the GI fluids. While drug solubility will define the upper limit of absorption, it can also affect the drug dissolution rate. A decrease in drug solubility in pediatric GI fluids can result in a lower amount of drug available for absorption, which might result in differences in oral bioavailability. To conclude on the therapeutic impact of these results, changes in drug solubility should be considered alongside the remaining factors that have the potential to affect absorption, including drug substance and formulation properties (i.e., dose administered, type of formulation, excipients present, and others), as well as physiological factors (i.e., gastric emptying, small intestinal transit times, intestinal surface area, and others). Age-appropriate in vitro drug dissolution studies with an appropriate pediatric dose and age-appropriate biorelevant dissolution conditions should also be performed in the future to further understand the impact of age-related factors not only on drug solubilization, but also on drug dissolution rate and extent. The incorporation of pediatric simulated GI fluid solubility and dissolution into physiologically based pharmacokinetic (PBPK) models offers the opportunity to mechanistically understand drug product behavior in vivo. 

## 5. Conclusions

Investigation of drugs’ solubility was performed for seven poorly water-soluble compounds in pediatric (i.e., representative of newborns and infants GI conditions) and adult biorelevant media. For the majority of the investigated compounds, drug solubility fell outside an 80–125% range from adult values in at least one of the developed pediatric media (five out of seven). Compounds with the highest lipophilicity (logP) showed the greatest solubility differences between adults and pediatrics as opposed to hydrophilic compounds, of which solubilities were not significantly affected by changes in media composition. Moreover, the importance of food composition and meal properties (e.g., fat and protein content cow formula vs. soy formula) on driving drug solubility was also demonstrated. From a clinical perspective, differences in drug solubility between adults and pediatric patients are one of the factors that can lead to changes in drug performance, such as deviations in the extent/rate of drug absorption of orally administered drug products and, therefore, may affect drug safety and efficacy. Solubility in newborns conditions was significantly different from infant conditions for eight out of the fourteen compounds investigated, demonstrating the importance of differentiating between the two subpopulations. Further investigation of intraluminal changes in pediatric populations must be undertaken to continue the development of representative pediatric GI fluids. This will allow the establishment of confidence in the pediatric biopharmaceutical methods discussed in this manuscript, which can be used to guide the development of age-appropriate medicines.

## Figures and Tables

**Table 1:** Physicochemical properties of the drugs used in this study, and respective chromatographic analytical conditions.

Compound	logP	pKa	MW (g/mol)	BCS Class	Column	Mobile Phase	Flow Rate (mL/min)	T (°C)	Injection Volume (µL)	λ (nm)	Run Time (Min)	HPLC Method Reference
Mesalazine	0.98 [8]	2.3 (acidic) and 5.69 (basic) [8]	153 [9]	IV [10]	^c^	MeOH: H_2_O with 0.01% TFA (5:95)	1	40	20	304	10	[8]
Montelukast	8.79 [11]	2.7 (basic) and 5.8 (acidic) [11]	586 [9]	II [11]	^b^	MeOH: Ammonium acetate buffer pH 5.5 gradient ^e^	1	20	100	284	15	[12]
Nitrofurantoin	−0.47 [9]	7.2 (acidic) [13]	238 [9]	II [14]	^b^	MeOH: Phosphate buffer 0.05 M pH 3 (40:60)	1	25	50	360	6.5	[15]
Naproxen	3.18 [9]	4.18 (acidic) [16]	230 [9]	II [17]		IACN: H2O with 0.1 % Formic acid (40:60)	1	40	100	239	6	[18]
Dipyridamole	2.74 [19]	5.7–6.4 (basic) [19,20]	505 [9]	II [20]	^b^	ACN: H_2_O with 0.1 % TFA (30:70)	1	25	50	284	8	[21]
Mebendazole	2.80	3.5 (basic)	295 [9]	II [9]	^a^	MeOH: Ammonium phosphate 0.05 M pH 5.5(70:30)	1	25	100	313	6	[22]
Amiodarone	7.57 [23]	8.73 (basic) [23]	645 [9]	II [17]	^d^	MeOH: Phosphate buffer 0.05 M pH3 (70:30)	1	25	20	241	5	[22]

**^a^** Waters Xbridge Shield RP_18_, 130Å, 150 × 4.6 mm, 3.5 µm. **^b^** Kromasil 100 Å C_18_ 250 × 4.6 mm 5 µm. **^c^** Luna C_18_ (2) 250 × 4.6mm 3 µm. **^d^** Agilent Zorbax 300SB C_8_ 3.5 µm 150 × 4.6 mm. **^e^** Gradient elution for Montelukast was the following, % buffer = 90 at t = 0 min; % buffer = 50 at t = 2 min; % buffer = 10 at t = 4 min; % buffer = 10 at t = 12 min; % buffer = 90 at t = 12.3 min.

**Table 2:** Mean solubility data ± standard deviation (SD) (µg/mL) in adult biorelevant media representative of GI conditions (*n* = 3).

Adult Biorelevant Media	Solubility (µg/mL)						
	Mesalazine	Montelukast	Nitrofurantoin	Naproxen	Dipyridamole	Mebendazole	Amiodarone
FaSSGF	3202.04 ± 119.20	0.85 ± 0.18	196.08 ± 11.73	30.65 ± 1.32	11524.19 ± 570.44	39.51 ± 4.45	18.82 ± 1.90
FeSSGF	1717.76 ± 392.37	-	389.56 ± 6.35	710.24 ± 26.65	158.27 ± 7.22	4.37 ± 0.90	-
FaSSIF-V2	2664.58 ± 145.74	8.28 ± 1.02	215.67 ± 1.96	1910.34 ± 94.91	11.86 ± 0.17	1.39 ± 0.16	24.88 ± 4.84
FeSSIF-V2	2882.02 ± 68.37	38.57 ± 9.15	209.40 ± 3.22	944.61 ± 42.83	81.38 ± 1.74	3.51 ± 0.17	652.19 ± 74.82

FaSSGF, adult fasted state simulated gastric fluids; FaSSIF-V2, adult fasted state simulating intestinal fluids; FeSSGF, adult fed state simulated gastric fluids; FeSSIF-V2, adult fed state simulated intestinal fluids.

## Data Availability

Data available on request.

## List of Abbreviations

**BCS**	Biopharmaceutics Classification System
**BSs and LC**	Bile salts and lecithin
**FaSSGF**	Adult fasted state gastric media
**FaSSIF-V2**	Adult fasted state intestinal media Version 2
**FeSSGF**	Adult fed state gastric media
**FeSSIF-V2**	Adult fed state intestinal media Version 2
**GI**	Gastrointestinal
**logP**	log (octanol/water partition coefficient)
**MW**	Molecular weight
**NaTC**	Sodium taurocholate
**P50%-FaSSIF**	Pediatric fasted state intestinal media formulated with bile salt concentrations 50% (i.e., 1.5 mM) of adult levels
**P150%-FaSSIF**	Pediatric fasted state intestinal media formulated with bile salt concentrations 150% (i.e., 4.5 mM) of adult levels
**pBCS**	Pediatric Biopharmaceutics Classification System
**PBPK**	Physiologically based pharmacokinetics
**Pi-FaSSGF**	Pediatric fasted state gastric media representative of infants (1–12 months)
**Pi-FeSSIF**	Pediatric fed state intestinal media representative of infants (1–12 months)
**Pn-FaSSGF**	Pediatric fasted state gastric media representative of newborns (0–28 days)
**Pnc-FeSSGF**	Pediatric fed state gastric media representative of newborns (0–28 days) fed cow’s milk-based formula
**Pns-FeSSGF**	Pediatric fed state gastric media representative of newborns (0–28 days) fed soy-based formula
**PLS-R**	Partial Least Squares Regression
**Q^2^**	Goodness of prediction
**R^2^**	Coefficient of determination
**SD**	Standard Deviation
**Sp/Sa (%)**	Pediatrics-to-adult solubility ratio
